# Detection of Peripheral Malarial Parasites in Blood Smears Using Deep Learning Models

**DOI:** 10.1155/2022/3922763

**Published:** 2022-05-24

**Authors:** Amal H. Alharbi, Aravinda C. V, Meng Lin, B Ashwini, Mohamed Yaseen Jabarulla, Mohd Asif Shah

**Affiliations:** ^1^Department of Computer Sciences, College of Computer and Information Sciences, Princess Nourah bint Abdulrahman University, P.O. Box 84428, Riyadh 11671, Saudi Arabia; ^2^N. M. A. M. Institute of Technology, Nitte 574110, Karkala, India; ^3^Ritsumeikan University, Kyoto, Japan; ^4^School of Electrical Engineering and Computer Science, Gwangju Institute of Science and Technology (GIST), Gwangju, Republic of Korea; ^5^Kebri Dehar University, Kebri Dehar, Ethiopia

## Abstract

Due to the plasmodium parasite, malaria is transmitted mostly through red blood cells. Manually counting blood cells is extremely time consuming and tedious. In a recommendation for the advanced technology stage and analysis of malarial disease, the performance of the XG-Boost, SVM, and neural networks is compared. In comparison to machine learning models, convolutional neural networks provide reliable results when analyzing and recognizing the same datasets. To reduce discrepancies and improve robustness and generalization, we developed a model that analyzes blood samples to determine whether the cells are parasitized or not. Experiments were conducted on 13,750 parasitized and 13,750 parasitic samples. Support vector machines achieved 94% accuracy, XG-Boost models achieved 90% accuracy, and neural networks achieved 80% accuracy. Among these three models, the support vector machine was the most accurate at distinguishing parasitized cells from uninfected ones. An accuracy rate of 97% was achieved by the convolution neural network in recognizing the samples. The deep learning model is useful for decision making because of its better accuracy.

## 1. Introduction

As per the World Health Organization, 3.4 million inhabitants in 92 countries may be at risk of malaria infection, with 1.1 billion people at high risk. Epidemiological factors can affect the transmission of malaria, including an ecological and epidemiological study performed in a confined, isolated location [1]. As a result, the WHO supports the advancement of rapid and inexpensive diagnostic testing that aids incorrect treatment method identification. In 2019, there has been an estimated 229 million cases of malaria worldwide [2, 3]. The rate of death totaled 409,000 cases of malaria, as measured by an annual survey [4]. Malaria is caused by parasites transmitted through mosquito bites from infected female Anopheles mosquitoes [5, 6]. In general, microscopy testing is widely accepted and widely used for attracting potential patients with malaria. Children under five are most likely to be affected by malaria [7, 8]. The WHO report predicts that the African region carries the highest share of the global malaria burden.

As per WHO research, the African region might feel the consequences of the global malaria burden [9]. Since it would provide more accuracy, improve consistency, and be cost effective in rural regions, an automated malaria diagnosis system would make a big difference in eliminating the shortage of this insufficiency [10, 11]. Thus, according to medical specialists from the World Health Organization, several malaria parasite groups may cause malaria infection in humans [12]. These include “Plasmodium Falciparum, Plasmodium Vivax, Plasmodium Malaria, Plasmodium Ovalle, and Plasmodium-knowlesi.” Out of these, the most common two classes are “ Plasmodium Falciparum and Plasmodium Viva.”

**Figure 1:**
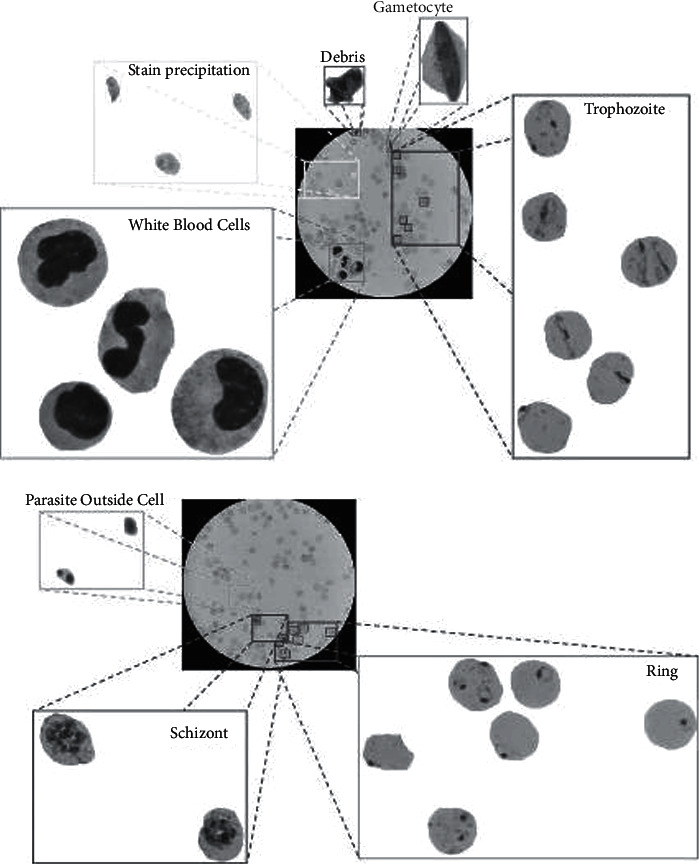
Stages of malaria in blood smear [13].

**Figure 2:**
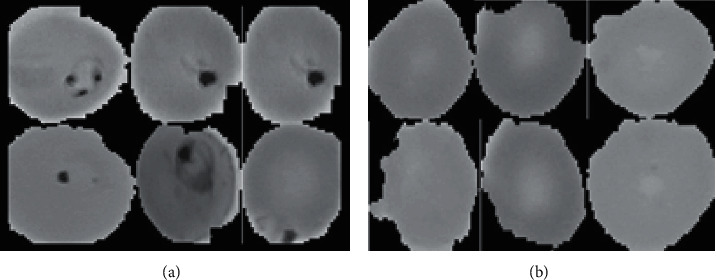
Affected samples and clean samples [14].

Figure 1 illustrates how malarial cells progress through their various stages: an examination of the first slide shows trophozoites and gametocytes of P. falciparum along with white blood cells as mentioned in Figure 2. A comparison is now made between the enlarged nucleus and the rest of the red blood cells. The second image proves how Plasmodium Falciparum is erected with Plasmodium Schizonts. There is a need for researchers to investigate innovations relating to malaria diagnosis to automate the process for society. This field has seen an increase in good research articles over several decades. Besides research articles, automatic malaria diagnosis has occurred with a variety of software tools and hardware tools.

## 2. Related Work

Makhija et al. implemented a V-value histogram method. It has achieved 60% sensitivity [1]. Rajaraman et al. designed the contrast enhancement and threshold-based segmentation approach. A qualitative analysis of two hundred patients' images was achieved using this method [2]. Suriya et al. worked on the color information-based pattern segmentation by considering 75 patients' image samples and achieved around 90% of detection [3]. Liang et al., according to their study, could detect 90% of the speckle noise images using a median filter based on histogram and morphological operations [4]. Vijayalakshmi et al. worked on microscopic images by applying transfer learning on VGG 16 and SVM classifier and claiming the result of 91% of classification accuracy [5].

Hommelsheim et al. implemented the DNA/RNA-binding domains with nucleotide sequence accuracy and redesigned transcription-activator-like signaling pathways to alter genomes with improved binding specificity [6]. Hawkes et al. worked on an analysis of rapid diagnostic tests and made the awareness of utilizing the RDT for equality and ease of use for cost effectiveness [7]. Ross et al. implemented a method for automating the diagnosis of malaria from thin blood smears that has been developed. Identification of infected erythrocytes is achieved with 75% sensitivity and an 85% positive predictive value (PPV) [8]. Das et al. worked on parasite characterization and classification using ML on light microscopic images of peripheral blood smears and achieved 89% classification using an SVM classifier [9].

Postiche et al. discussed the development of image analysis and machine learning for the diagnosis of malaria microscopically as well as the emergence of smartphone technology for future diagnosis [10]. LeCun et al. worked to find complex structures in large datasets, and the backpropagation algorithm was implemented to propose that a machine could constantly update its internal parameters based on its representation in the prior phases. [11]. Dahou Yang et al. compared the detection of antimalarial efficacy of an image-based cytometer with a commercial flow cytometer and the results of these two tests [12].

Yunda et al. determined that by applying principal component analysis to samples of thick film blood, they could reduce the number of features. [15]. Kaewkamnerd et al. to detect the sensitivity of plasmodia on thick blood films, created a two-stage algorithm [16]. Hanif et al. developed an elongating technique for the enhancement and segmentation of heavy blood smear images of Plasmodium falciparum. They reduced the amount of noise and blurring while increasing the contrast range to make the images more visible [17]. The existing models suffer from various issues such as overfitting [13, 18, 19], vanishing gradient [20–22], and poor convergence speed [23–25] kinds of problems.

## 3. Overview of Convolution Neural Network (CNN)

**Figure 3:**
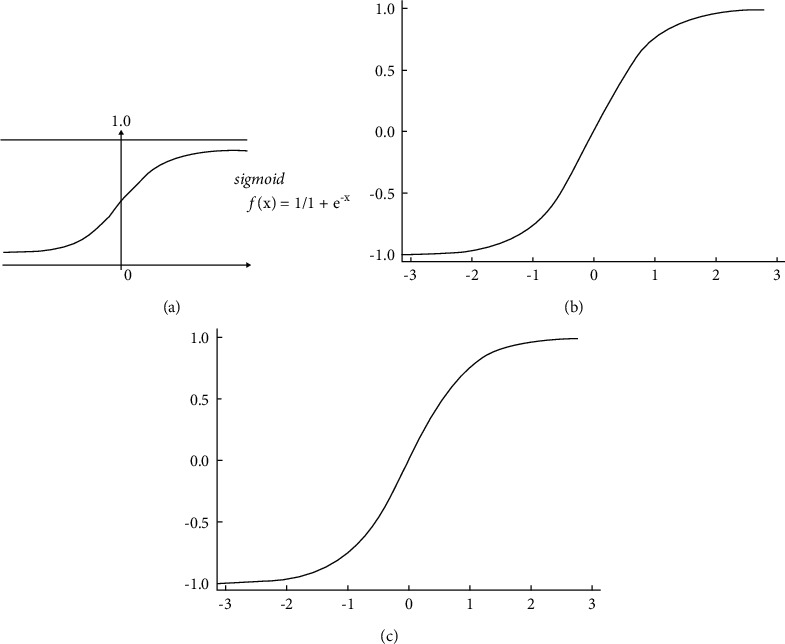
Example of a two-part figure with individual subcaptions showing that the captions are sigmoid nonlinearity range between [0, 1] and tanh nonlinearity squashes real numbers to the range between [−1, 1]and ReLU is which is zero when *x* < 0 and then the linear with slope 1 when *x* > 0. (a) Sigmoid, (b) Tanh, and (c) ReLU.

Convolution layers comprise the CNN (convolutional neural network). Pixel-by-pixel weights and biases are learned. The synapse cell accepts a variety of inputs before combining the weights, either from the input in each layer or from the weights and biases passing through the activation node. This level comprises the input pixels and weights that are shared by the layer's neurons [26–28]. Furthermore, the inputs to standard neural networks are vectors of a single dimension, but in CNN, they are represented as multichannel images. It uses the framework of this algorithm to optimize such algorithms as random gradient, Adam, AdaGrad, etc. as well as to perform tasks such as object detection, image classification, and localization as mentioned in Figure 3.

### 3.1. Pooling Layers

Filters are applied to the input images to generate feature maps that distinguish the inclusion of those features and they accomplish it by pooling layers. The constraint of this feature map is that it will show a feature's location precisely. The feature map has the dimensions *nh∗nw∗nc*, and the output obtained after the pooling layer is (*nh* − *f*+1)/*s∗*(*nw* − *f*+1)/*x∗nc*. There are three types of pooling layers such has, Max, Averaging, and Global Pooling. Feature pooling takes the maximum value from a region on the feature map. Average pooling takes the average of the elements present in the neighborhood. The Global pooling takes the value of the entire feature map and scales it down.

### 3.2. Frequently Used Activation Layer

  Sigmoid: the mathematics of the sigmoid function involves the outcome of taking a single number and achieving convinced mathematical operations. *σ* (*x*) = 1/(1 + *e*^−*x*^). This takes the real input number and flattens the range between 0 and 1.
  Tanh: in the study, tanh is a nonlinearity that is preferred over the sigmoid functions. Even though it shows better results than the sigmoid functions, it is still not possible to solve gradient problems using the Tanh function. tanh(*x*)=2*σ*(2*x*) − 1.
  ReLU: as said in the function, the activation is simply a threshold at zero. Tanh functions are mathematically portrayed as *f*(*x*)=max(0, *x*).

## 4. Methodology

Various examination of the freely accessible datasets was utilized for classification, augmentation, and preprocessing. As shown in Table 1, a few authors have already provided a dataset that we evaluated for collection, classification, augmentation, and preprocessing techniques.

### 4.1. Datasets

Figure 2 illustrates two sets of datasets consisting of roughly 13,000 samples, one parasitized and one nonparasitic, illustrating infected and uninfected malarial blood samples.

### 4.2. Classification of Malaria Cells

The use of computer vision and machine learning algorithms to diagnose malaria has recently got performance metrics in plenty of new studies. As part of a collaborative effort, a recently proposed automated system for detecting and acting on red blood cells was recently presented.

### 4.3. Image Smoothing

**Figure 4:**
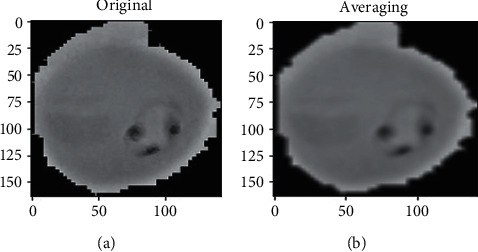
Two parts with separate captions for smoothing the original infected sample and smoothing averaging the original infected sample. (a) Infected original sample. (b) Image greyscale.

**Figure 5:**
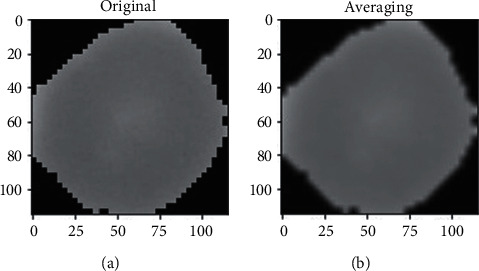
Two parts with separate captions for smoothing the uninfected original sample and smoothing averaging the original sample. (a) Infected original sample. (b) Infected original sample.

The impact of blurring was applied to several smoothing algorithms, such as Gaussian noise, salt pepper noise, and bilateral filters for both noisy images, and this was compared using the Gaussian, median, and bilateral filters. By eliminating noise and blurring an image, 2D convolution filtering utilized in various low-pass and high-pass filters achieved promising results. The high-pass filter recognized the edges in a cell image and generated promising results. For this cell image, a 2 averaging filter kernel has been used, *K* = 1/9 as shown in Figures 4 and 5.

### 4.4. Gabor Filtration Technique

**Figure 6:**
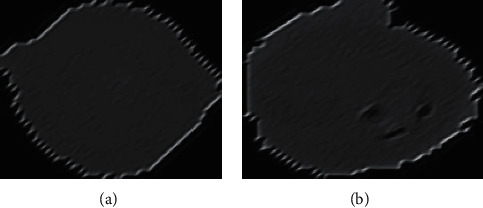
Infected- Gabor-filtered and the other uninfected Gabor-filtered. (a) Affected initial sample. (b) A greyscale sample.

The presence of no malaria-infected cells in many samples allows them to be used as a method to reduce the overall processing run time, which is why statistical analysis is applied to calculate the infected cell sample numbers. A threshold was applied using the Gabor filter method to color the image of the infected area after this was noticed. Distortion was detected in both the background and inside RBCs, according to results of this approach. Later, morphological series have been used to fill the gaps to gain distinct samples, as illustrated in Figure 6. The precision of a Gabor filter influences its orientation; this has kernels that are common to the 2D field and depict essential spatial localization and orientation aspects; as a result, the kernels of this filter are also relative term of dimensions mentioned in (1).

(1) $$\begin{array}{c} \psi_{\omega \phi }=\frac{\omega }{\sqrt{2\pi C}}\frac{e^{-}\omega^{2}\left(4ap^{2}+bq^{2}\right)}{\left(8c^{2}\right)\left(e^{i}a\omega -e^{-}c^{2}/2\right)}. \end{array}$$

The actual and imagined regions of the filtration are shown in Figure 4. (infected) and Figure 5. (uninfected). We assume that the value of I(ap + bq) is a grey value at (ap, bq). The sample convolution and the scale's Gabor kernel, as well as the direction of *θ*, are specified as

(2) $$\begin{array}{c} \;G_{\omega ,\phi }=I⊗\psi_{\omega }\phi . \end{array}$$

Equation (3) now has two actual and imagined values. Each separated by a distance orientation's response is specified as

(3) Iωθz=ReG,ω,θz2+ImG,ω,θz2.

**Figure 7:**
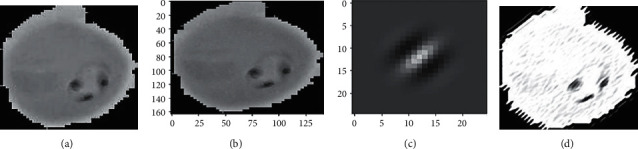
Infected kernel sample, infected greyscale, and an output image that is resized according to its kernel size. (a) Infected sample, (b) Gray scale, (c) Gabor filter image, (d) Output generated.

Figures 6 and 7 illustrates images combined with subsampled values analyzed with Gabor. The values considered *k*-size = 20^*∗*^20, *σ*=4, *θ*=1^*∗*^*np*.*pi*/2, lamda(*λ* )=1^*∗*^*np*.*pi*/4, *ϕ*=0.8.

### 4.5. Data Preprocessing

The model's actions depend on the data that were provided in the supervised process of learning. As a result, it has a profound influence on decision making. A large dataset is obtained because the image smoothing algorithm is applied for feature extraction. The vector was standardized in the range of 0 to 255 as the first step toward accurate data identification. The chi-square feature selection method was used for selecting 80% of the most useful features for the final vectors.

## 5. Classifications

### 5.1. Support Vector Machine

A support vector machine (SVM) is useful for deciding the optimal location of a decision boundary or for learning statistics and for determining when it is necessary to separate classes. Regarding multiclass classification problems, this study used the “each” strategy, wherein labels are assigned from a finite set of several elements. Infectious parasite samples and unaffected parasitic samples are two classes that were used to estimate the number of classes. The “one-to-one” classifier results can be transformed to form a decision function of shape by using the decision function shape option, (13000samples, 2classes). The test vectors are analyzed by applying each classifier to them, giving each vote.

### 5.2. XG-Boost Algorithm

Gradient boosting reduces the loss function by building models from single weak learners in an iterative fashion instead of building a complete model from random subsets or features like the random forest. The loss function for gradient boosting is minimized using gradient descent. Based on the application of this algorithm, it is possible to achieve a score of about 90% in accuracy and speed because it uses advanced regularization techniques to prevent overfitting and speed up computation, as shown in Table 2.

### 5.3. Proposed Convolutional Neural Network

**Figure 8:**
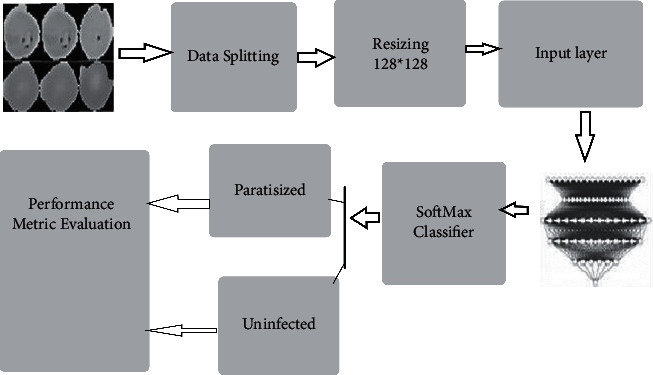
Proposed block diagram of CNN.

**Figure 9:**
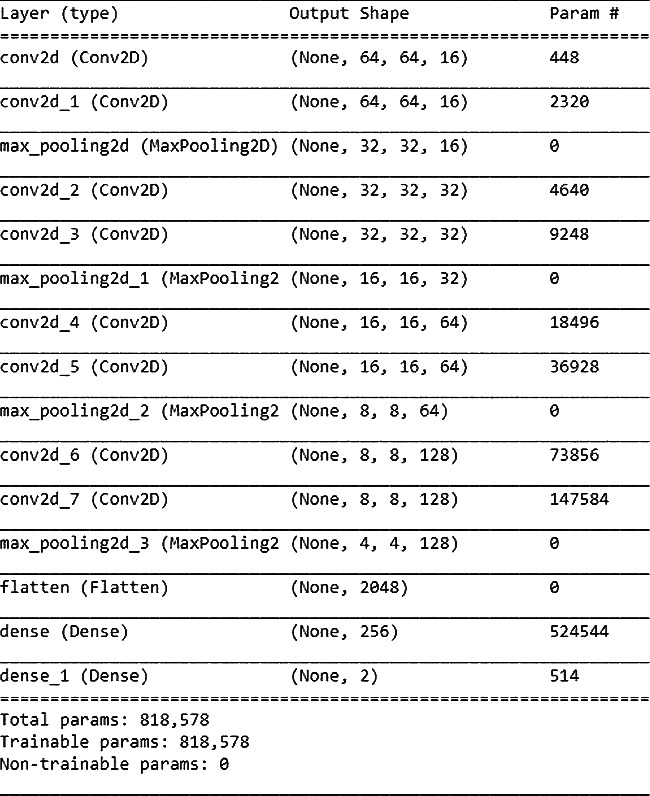
Architecture configuration set-up.

A trainable customizable network, which is divided into four fully connected layers, is based on the convolution layer, the pooling layer, and the average pooling step to obtain features, reduce computations, and update features. 26,188 samples were divided into three sets: training, testing, and validation. It was then converted to a 128 × 128 pixel size. According to Figures 8 and 9, the study included 13,779 uninfected samples.

## 6. Evaluation Metrics

### 6.1. Optimizer Function

In contrast to the RMSProp optimizer function, the Adam optimizer could much more successfully overcome the AdaGrad optimizer's inadequacy. It is a much more advanced version of stochastic gradient descent wherein the weights are continuously updated with the training data. The iterations must be stated in Step 1 for the algorithm to work. The very first step is to get a gradient. Step 2 is by using the moving averages formula for determining the moving averages. In this step, the estimator's bias is corrected, where *xhat* and *yhat* are biased correction equations, and in the last step of the Adam algorithm, the weights are adjusted in the network using the z-expression.

### 6.2. Loss Function

In the optimizer, the neural net's weights and biases are updated to reduce the loss function. Loss functions are defined as ways of mapping input functions to output functions. This function will decide the probability value of a prediction class by calculating the categorical cross-entropy, as shown in (4).

(4) $$\begin{array}{c} L\left(\hat{y,y}\right)=-\frac{1}{N}\frac{N}{in}\frac{ca}{ca=1}1yi_{\varepsilon }C_{ca}\log\;P_{m}\left[y_{i}\varepsilon c_{ca}\right], \end{array}$$

where “ca” is the number of categories and “in” is the number of observations.

### 6.3. Performance Analyses

#### 6.3.1. Experimental Set-Up and Performance Metrics

On the server, the recognition system is installed for online access. The CPU is a Lenovo ThinkPad workstation, RAM is 32G, and the operating system is Windows 10.

The problem consists of sorting the images into two classes: parasitized (i) and uninfected (ii). The *F*1-score is calculated by using the harmonic mean of precision and recall. It can be evaluated as mentioned in (5).

(5) $$\begin{array}{c} F1-score=2*\frac{\left(\mathrm{precision\times recall}\right)}{\left(\mathrm{precision+recall}\right)}. \end{array}$$

True positive rate is another well-known measure. Compared to actual positives correctly classified as positives, this reflects the number of true positives, and it can be defined as mentioned in (6).

(6) $$\begin{array}{c} \text{True Positive Rate }=\frac{TP}{\left(TP+FN\right)}. \end{array}$$

Accuracy can be defined as the systematic errors that measure the differences between true and predicted values, and it can be defined as mentioned in (7).

(7) $$\begin{array}{c} \mathrm{Accuracy=}\frac{\left(\mathrm{TP+TN}\right)}{\left(P/N\right)}. \end{array}$$

The positive predicted value represents the subjects who were positive for the presence of the disease during the screening procedure, and it can be defined as mentioned in (8).

(8) $$\begin{array}{c} \mathrm{PositivePredictedValue=}\frac{\mathrm{TP}}{\left(\mathrm{TP+FP}\right)}. \end{array}$$

The negative predicted value represents how likely it is for the subjects screened for the disease to have a negative result, and it can be defined as mentioned in (9).

(9) $$\begin{array}{c} \text{False Positive Rate }=\frac{FP}{\left(FP+TN\right)}. \end{array}$$

The false negative rate can be calculated by adding the number of positive events that were misclassified as negatives to the total number of positive events, and it can be defined as mentioned in (10).

(10) $$\begin{array}{c} \text{False Negative Rate }=\frac{FN}{\left(FN+TP\right)}. \end{array}$$

**Figure 10:**
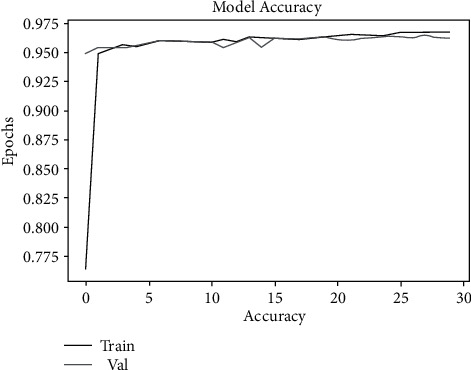
Training and validation accuracy.

**Figure 11:**
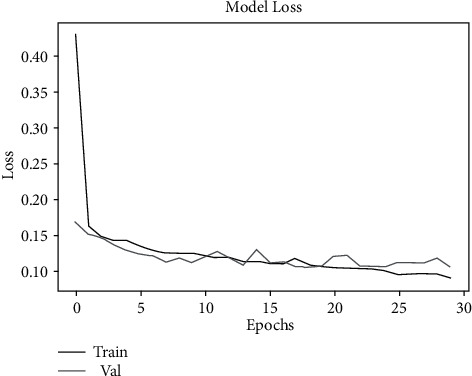
raining and validation loss.

**Figure 12:**
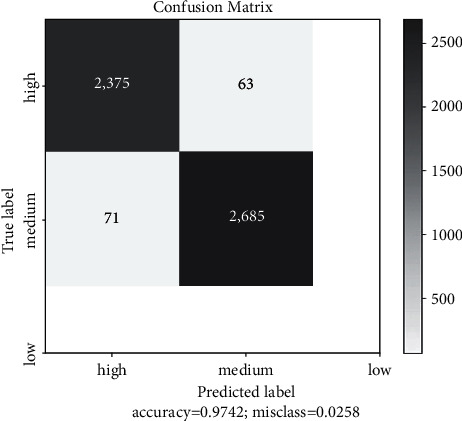
Confusion matrix accuracy 97%.

**Figure 13:**
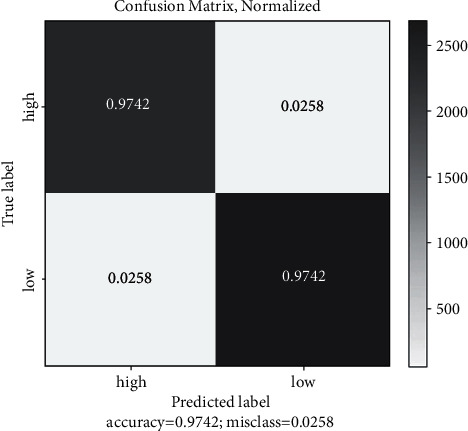
Confusion matrix normalized.

**Figure 14:**
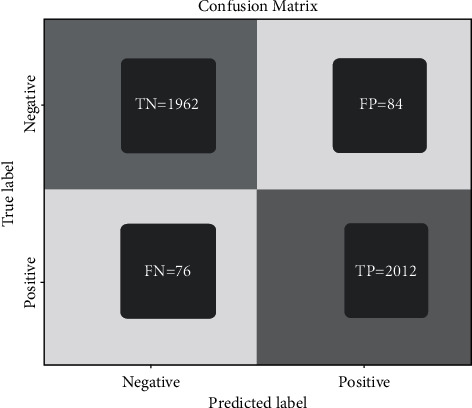
Confusion matrix true positive and true negative.

To determine the accuracy of malarial parasite detection using ML algorithms, experiments were conducted with several algorithms. Based on Tables 2 and 3, the classification accuracy obtained by the SVM was around 94%, and XG-Boost had an estimated classification accuracy of 90%. As shown in Table 4, an estimated 98.0% of the data was able to be classified by the convolution neural network. Figures 10 and 11 show the training and validation loss followed by training and validation accuracy, respectively, and Figure 12 the confusion matrix accuracy, Figure 13 confusion matrix normalized, and Figure 14 confusion matrix true positive and true negative, respectively.

## 7. Conclusion

Plasmodium parasites cause dengue infection in red blood cells. Counting blood samples mechanically is a very time-consuming process that results in a tedious diagnosis strategy. For the detection and analysis of this malarial virus, the XG-Boost classification algorithm, support vector machine, and neural network algorithms were compared using Gabor filters. Convolutional neural networks perform well on the same datasets when analyzing and recognizing them. A model is developed that analyzes the blood sample to determine parasitized or uninfected cells. Using this model, we aim to reduce model discrepancies and improve their robustness and generalization. We collected 13,750 parasitized samples and 13,750 nonparasitic samples for comparative analyses. Support vector machines were accurate to 94%, while XG-Boost achieved 90% accuracy, and neural networks achieved 80% accuracy, respectively. Both parasitized and uninfected cells were more accurately classified by the support vector machine than by the other two models. In either case, convolutional neural networks were designed to recognize the samples with 97% accuracy. In terms of decision making, these models are helpful because of their improved accuracy.

## Figures and Tables

**Algorithm 1:**
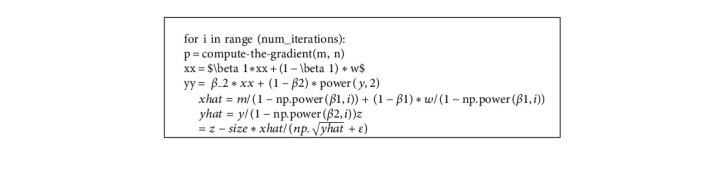
Adam optimizer algorithm.

**Table 1:** Existing study of methodologies.

Model	Methodology
Rajaraman et al. [2]	CNN with 2-level segmentation
Narayanan et al. [29]	Encoder-decoder architecture
Tran et al. [9]	Segmentation using deep learning
Li et al. [30]	Deep CNNs for HEp-2 cell classification
Das et al. [31]	Bayesian learning and support vector machine

**Table 2:** 94% accuracy in support vector machine classification.

Precision measure	Recall measure	*F*1-score (%)
Parasitized-results-0.9259	0.9615	0.9433
Infected-results-0.9565	0.9166	0.9361

**Table 3:** 90% accuracy of extreme gradient boosting algorithm classification.

Precision measure	Recall measure	*F*1-score (%)
Parasitized-results-0.8275	0.9230	0.8727
Infected-results-0.9047	0.7916	0.8444

**Table 4:** Proposed CNN with state-of-the-art models, 96%.

Authors work	Methodology	Accuracy in %	Sensitivity	Specificity	*F*1-score
Rajaraman et al. [2]	Pretrained-CNN	0.98	0.981	0.992	0.987
Vijayalakshmi et al. [5]	CNN with 2-level	0.977	0.971	0.972	0.959
Liang et al. [4]	CNN (16 layers)	0.973	0.969	0.977	—
Das et al. [9]	Bayesian learning	0.84	0.981	0.689	—
Ross et al. [8]	Image enhancement	0.73	0.85		
Proposed model	Customized CNN	0.98	0.985	0.988	0.987

## Data Availability

The data used to support the findings of this study are available from the corresponding author upon request.
